# Enhanced Functional Connectivity Within Executive Function Network in Remitted or Partially Remitted MDD Patients

**DOI:** 10.3389/fpsyt.2020.538333

**Published:** 2021-01-28

**Authors:** Yuchen Wang, Aixia Zhang, Chunxia Yang, Gaizhi Li, Ning Sun, Penghong Liu, Yanfang Wang, Kerang Zhang

**Affiliations:** ^1^Department of Psychiatry, First Hospital of Shanxi Medical University, Taiyuan, China; ^2^Department of Medical Psychology, College of Humanities and Social Science, Shanxi Medical University, Taiyuan, China; ^3^College of Nursing, Shanxi Medical University, Taiyuan, China

**Keywords:** major depressive disorders, executive functions, fMRI, therapy, persistent cognition impairment

## Abstract

**Background:** Impaired executive function (EF) is associated with a range of typical clinical characteristics and psychosocial dysfunction in major depressive disorder (MDD). However, because of the lack of objective cognitive tests, inconsistencies in research results, and improvement in patients' subjective experience, few clinicians are concerned with the persistent impairment of EF in euthymia. The study makes a further investigation for EF in remitted and partially remitted MDD patients *via* multiple EF tests and fMRI, so as to explore the executive function of patients in euthymia.

**Methods:** We recruited 19 MDD patients and 17 age-, gender-, and education-matched healthy controls (HCs). All participants completed EF tests and fMRI scanning. Bilateral dorsolateral prefrontal cortex (dlPFC) regions were selected as the region of interests (ROIs) to conduct seed-based functional connectivity (FC). We conducted fractional amplitude of low-frequency fluctuations (fALFF) analysis for all ROIs and whole brain.

**Results:** All MDD patients were in remission or partial remission, and they were comparable with HCs on all the EF tests. MDD group showed increased positive FC between left dlPFC and cerebellar Crus I, right dlPFC and supramarginal gyrus after 8-weeks treatment, even taking residual depressive symptoms into account. We did not find group difference of fALFF value.

**Conclusion:** MDD patients persisted with EF impairment despite the remission or partially remission of depressive symptoms. Clinicians should focus on residual cognitive symptoms, which may contribute to maximize the efficacy of routine therapy.

## Introduction

Major depressive disorder (MDD) is a major contributor of global burden of diseases (1), in which cognition deficits is a core symptom (2). Research has identified that patients with MDD have a broad range of cognition impairments, which still exists even after the remission of clinical symptoms (3), particularly the impairment of executive function (EF) (4–6). EF is involved in the generation and maintenance of goal-directed behavior by planning, decision making, inhibition control, etc (7). Varieties of cognitive functions, such as memory, attention, and problem-solving, depend heavily on different dimensions of EF (8). Increasing evidences have indicated that impaired EF, particularly inhibition as the key component, is associated with a series of typical depressive characteristics: rumination (9, 10), negative attentional bias (11), emotion regulation dysfunction (12), etc. Likewise, impaired EF is closely related to psychosocial dysfunction in multiple domains such as occupational function, which contribute to huge social cost (13, 14). Furthermore, EF is an available predictor for treatment outcomes (15). Therefore, it is necessary to focus on and treat impaired EF for MDD patients. However, published literature have not come to an agreement on whether or not current antidepressant could improve EF.

Some behavior-level studies suggest that antidepressant could improve the impaired EF of MDD patients (16, 17). On the contrary, recent meta-analysis indicated that EF impairment persisted despite a clinical remission (4, 5). However, it is insufficient and difficult to illustrate the effect of antidepressant on EF only *via* behavior tests considered the fact that some fMRI studies recorded the abnormal neural activity in MDD patients in the case of their cognitive performance comparable to healthy controls (18, 19). Researchers explain the phenomenon as neurocompensatory effects (20, 21): behavioral performance of psychiatric patients could achieve the same level as healthy controls (HC) by increasing activity of neural underpinnings of cognition. But some researchers reported abnormal neural activity concurrent with behavioral impairment in patients with mental disorders (22, 23), which suggests that the range of neurocompensatory processes is limited and it is also a representation of EF impairment. Therefore, it is necessary to address the neural activity associated with EF besides cognition tests when assessing the improvement of EF after treatment. However, the results of fMRI studies are inconsistent. Wagner et al. found that antidepressant normalized abnormal activation associated with EF (24). Smith et al. showed that remitted MDD patients were comparable to healthy controls on EF tests and neural activity underlying EF (25). Bartova et al. showed reduced deactivation of default mode network (DMN) during working memory tasks and increased connectivity between DMN and salience network (SN) in remitted MDD patients (26). Importantly, however, the fMRI studies we demonstrated above measured EF through single task. EF is a complex construct which includes at least shifting, inhibition, working memory, updating, verbal fluency, and planning (6). One-sided assessment influences the location and direction of activation difference, and limits our holistic understanding of the effect of antidepressant on EF. Furthermore, any of mental functions, especially those advanced mental functions like EF, is carried out by network consisting of multiple synergistic brain regions. Whereas, we know little about the functional connectivity (FC) within network underlying EF in remitted MDD patients. Thus, we aimed to make a further investigation to better characterize the nature of neural basis of EF in remitted MDD patients.

Dorsolateral prefrontal cortex (dlPFC) plays an important role in EF. Previous studies have shown that functional impairment of dlPFC in MDD patients is closely related to cognitive dysfunction (27). Synder reviewed that dlPFC is involved in each domain of EF (6). Furthermore, dlPFC is also a key node of frontoparietal network (FPN) proposed by Dosenbach et al. (28). According to Dosenbach, FPN occupies in top-down control and provides rapid control initiation and adjustment signals for other brain networks, in which dlPFC exerts adjust control through carrying error-related activity. The main purpose of present study is using fMRI, a powerful non-invasive neuroimaging technique to explore the FC within dlPFC-based EF network during resting state combined with multiple EF tests in remitted MDD patients. Previous fMRI studies have provided rich evidence that emotional, cognitive, and behavioral impairments in depression are associated with functional abnormalities in specific regions and connections (29). Based on this, we evaluated the activity intensity of dlPFC as well. We hypothesized that MDD patients persist altered FC and/or activity in neural basis underlying EF despite the remission of depressive symptoms.

## Methods

### Participants

We recruited MDD patients from the out-patient and in-patient in the Department of Psychiatry, First Hospital of Shanxi Medical University. MDD diagnosis was determined by two practiced psychiatrists according to the diagnose criteria of MDD in Diagnostic and Statistical Manual of Mental Disorders Fourth Edition (DSM-IV). Further inclusive criteria for MDD patients: (1) total score on the 17-item Hamilton Rating Scale for Depression (HAMD-17) ≥ 17; (2) aged between 18 and 55 years. The exclusion criteria include: (1) left-handedness; (2) comorbidity with or a history of other DSM-IV Axis I disorders; (3) contraindication of MRI scanning; (4) history of neurological diseases, severe medical illness, etc. All participants take fluoxetine at a dose 20 mg−40 mg/d for 8 weeks. We assessed the clinical characteristics of patients again after treatment. Only those patients who completed all tests we need both at pretreatment (week 0) and posttreatment (week 8) were included. In addition, one participant was excluded due to excessive head motion (exceed 2.5 mm or 2.5° in any direction or angular). Finally, 19 patients were included in our analysis.

We also enrolled 17 healthy controls (HCs) well-matched with MDD patients in age (18–55), gender and years of education. HC did not meet any criteria of Axis I disorders and personality disorders, and the score of HAMD-17 is <7. Additional exclusion criteria include: (1) left-handedness; (2) contraindication of MRI scanning; (3) history of neurological diseases, severe medical illness, etc. The study was approved by the Ethical Committee of the First Hospital of Shanxi Medical University. All participants signed the informed consent.

### Assessment

#### HAMD-17

The scale is a most commonly used tool to quantify the severity of depressive symptoms of patients in the last week. In the study, HAMD-17 was administered by consultant psychiatrists.

#### Executive Functions

We assessed EF by the following tests: Stroop Color Word Test (SCWT; for inhibition), Digital Span Tests (DST; for working memory), Wisconsin Card Sorting Test (WCST; for shifting), and Semantic Verbal Fluency Test (SVFT; for verbal fluency). SCWT consists of three 30-items subtests: word task (reading words of color), color task (naming color of color circles), and color-word task (identifying the color ink of printed color words). Relative reaction time (RRT; the difference reaction time between color-word condition and color-naming condition) and accuracy in color-word condition reflect the ability of inhibition. DST asks participants to repeat the numbers in the same (Forward DST) and reverse order (Backward DST) as presented. The sequence length will increase if participants give the correct order, or we'll present another sequence of numbers. The test will finish when participants failed twice. Total length of sequence given correct answer reflects the working memory span. WCST asks subjects to sort cards to specific dimension according to feedback. If correct matches achieve a fixed number, the classification rules will change. We evaluate shifting ability by response administered (RA), categories completed (CC), correct response (RC), errors response (RE), perseverative errors (PE), and non-perseverative errors (nPE). In SVFT, participants need to say as many as they can in 1 min from certain semantic categories (fruits and vegetables). Total number of fruits and vegetables they say within a given time represent the ability of verbal fluency. All of the tests were administered by senior psychologists.

### Image Analysis

#### Acquisition

Image was performed by a Simens TrioTim 3.0T scanner with 12-channel birdcage head coil at the Shanxi Provincial People's Hospital. T1-weight anatomical images was used to acquire anatomical MRI data with following parameters: repetition time (TR) = 2,300 ms, echo time (TE) = 2.95 ms, flip angle (FA): 90°, field of view (FoV): 225 × 240 mm, matrix = 240 × 256, 160 volumes. Rest state fMRI data was acquired with echo-planar imaging (EPI) pulse sequence. Parameters are as follows: TR = 2,000 ms, TE = 30 ms, FA = 70°, FoV = 24 × 24 cm, matrix = 64 × 64, slice thickness = 3 mm, measurements = 212.

#### Preprocessing

Image preprocessing was conducted by DPARSF V4.4, a data processing assistant for resting-state fMRI developed by Yan et al. (30). Preprocessing steps are as follows: (1) removing first 10 time points; (2) slice timing; (3) realignment; (4) nuisance covariates regression (liner trend, Friston-24 head motion parameters, global signal, white matter, and cerebrospinal fluid signal); (5) normalizing to the Montreal Neurological Institute (MNI) space, resample to 3 × 3 × 3 mm^3^ voxels; (6) smooth with 4-mm FWMH Gaussian kernel; (7) filter (0.01–0.1 Hz). We made global signal regression (GSR) for getting more reliable and accurate data. Several advantages of GSR have been reported including closer relationship to DTI-based anatomy, better delineation of subcortical nuclei, improved specificity of positive correlations, and removal of motion, cardiac and respiratory signals known to correlate with the global signal (31). We calculated the mean framewise displacement (FD) to evaluate the head motion. The FD threshold is 2.5 mm and 2.5°. The head motion exceeded 2.5 mm or 2.5° in any direction or angular would be excluded in our analysis (One participate is excluded in the fMRI analysis). The FD was calculated for all resting state volumes (32). The mean FD is derived from Jenkinson's relative root mean square (RMS) algorithm (33). We also used New Segment function in DPARSF to calculate gray matter volume, which was used as a covariate later.

#### Functional Connectivity Analysis

According to the coordinate proposed by Dosenbach et al. (28), region of interests (ROIs; bilateral dlPFC, separate) were generated as 6-mm radius spheres using the WFU_Pickatlas 3.0.5. Seed-based FC was used to calculate the FC between ROIs and whole brain. A Fisher's *Z* analysis was used to transform correlation coefficient values to z-score.

#### fALFF Analysis

fALFF (fractional amplitude of low-frequency fluctuations) is an effective index to reflect the intensity of spontaneous neural activity for resting state fMRI study (34). We conducted fALFF analysis for all ROIs and further conducted an exploratory whole-brain analysis to explore brain activity associated with EF.

### Statistics Analysis

Demographic and clinical data analysis were conducted by SPSS 18.0. Chi-square test was used for categorical variables and *T*-test was used for continuous variables. We did not conduct the correction for multiple comparisons across statistical tests on neuropsychological performance. We paid more attention on the posttreatment patients and HCs rather than these three groups.

For fMRI data, we make a contrast for FC and fALFF after treatment of patients with HC by DPABI. To avoid the effect of anatomical variation (35, 36), we added gray matter volume as covariates. We also added potential confounded factors (HAMD score and head motion were added in the study) as covariates. Gaussian Random Field (GRF) Theory correction was used for multiple comparison correction with voxel *p* < 0.001.

## Results

### Demographic and Clinical Characteristic

No significant difference was found between patients who was included in analysis and those excluded from analysis in age, gender, years of education, age of first onset, symptom severity, SCWT, DST, WCST, and SVFT (all *p*s > 0.05). There was also no significant difference between MDD patients and HC in age, gender, and years of education. MDD patients scored lower than HC in SCWT and SVFT at baseline, but these differences disappeared at 8 weeks. Compared to baseline, MDD patients showed a significant reduction in HAMD-17 score and all patients were in remission or partial remission (particularly, 11 patients were in remission and 15 patients showed a reduction in HAMD more than 50%). But, MDD patients still had a higher HAMD-17 score compared to HC after treatment. Compared to baseline, MDD patients had a significant improvement in RC and RE of WCST, and showed a trend to improvement in SVFT, errors of SCWT, and CC in WCST (see Table 1).

**Table 1:** Demographic and clinical characteristic of MDD and HC.

	**MDD**		**HCs (*M ± SD*)**
	**Pretreatment** **(*M ± SD*)**	**Posttreatment (*M ± SD*)**	
Male/Female	8/11	-	9/8
Age	31.95 ± 9.13	-	30.47 ± 8.50
Education	12.00 ± 3.38	-	11.76 ± 1.56
HAMD-17	21.37 ± 3.08^***^^###^	7.26 ± 3.02^***^	0.76 ± 0.66
Onset Age	30.58 ± 9.31	−	−
DST	14.63 ± 2.36	14.63 ± 1.80	14.76 ± 2.08
SVFT	16.58 ± 4.22^*^^#^	18.16 ± 3.27	19.71 ± 4.27
**SCWT**			
Errors	0.95 ± 1.35^*^^#^	0.32 ± 0.75	0.18 ± 0.728
RRT	14.74 ± 5.89	14.89 ± 7.31	13.88 ± 6.41
**WCST**			
CC	2.32 ± 2.19^#^	3.42 ± 2.22	3.18 ± 2.43
RA	125.21 ± 7.45	123.79 ± 8.97	121.24 ± 10.20
RC	60.00 ± 18.66^##^	69.74 ± 20.40	60.18 ± 10.14
RE	65.21 ± 22.46^##^	54.05 ± 24.60	55.06 ± 25.15
PE	49.21 ± 22.17	40.60 ± 26.00	38.41 ± 24.84
nPE	18.58 ± 5.15	18.58 ± 7.88	16.65 ± 6.06

* *p < 0.05 (MDD vs. HC)*;

*** *p < 0.001 (MDD vs. HC)*;

# *p < 0.1 (pretreatment vs. posttreatment)*;

## *p < 0.05 (pretreatment vs. posttreatment)*;

### *p < 0.001 (pretreatment vs. posttreatment)*.

### fMRI Data

**Figure 1:**
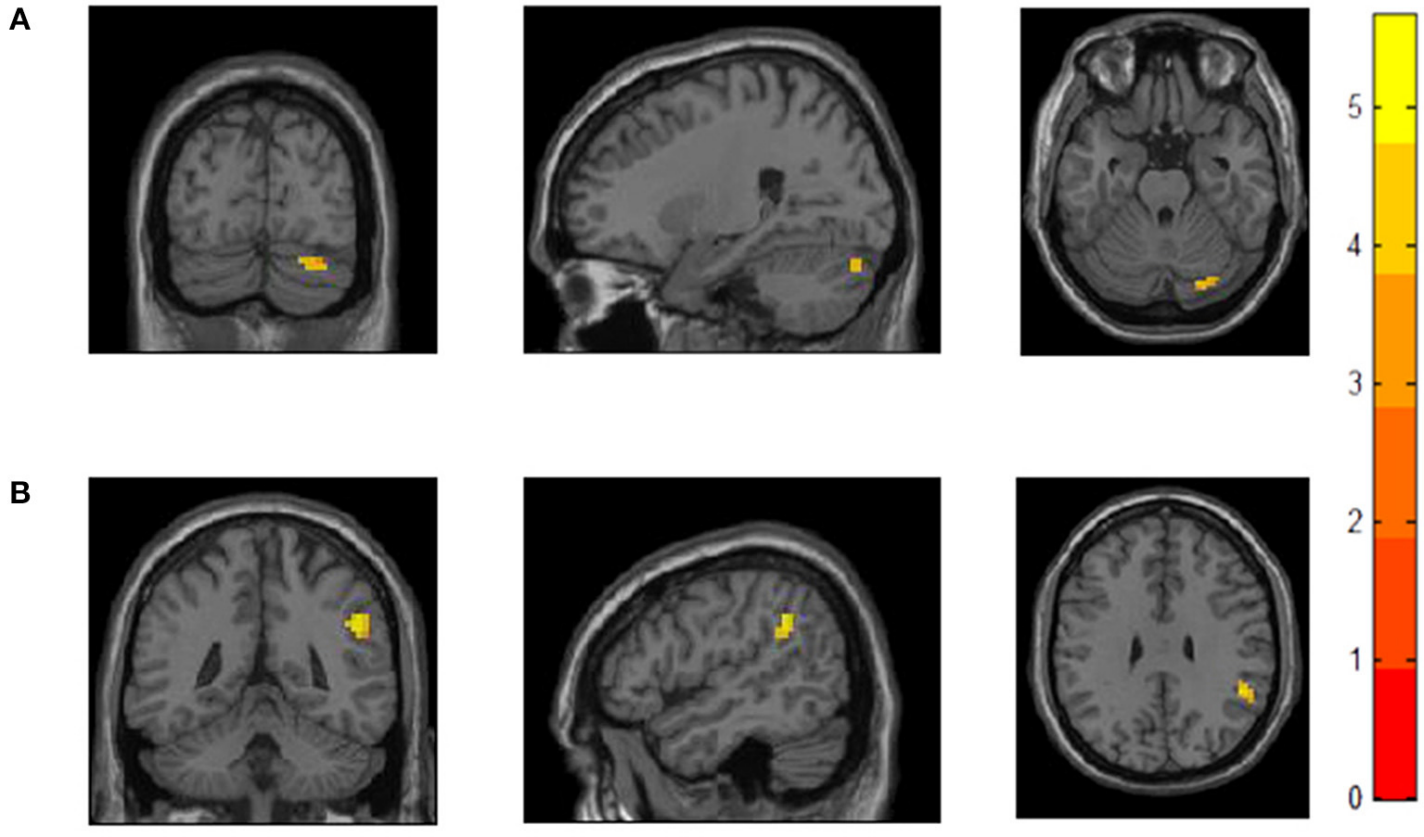
Increased functional connectivity with bilateral dlPFCin MDD patients compared to HC. **(A)** MDD patients showed increased FC between left dlPFC and right cerebellar Crus I. **(B)** MDD patients showed increased FC between right dlPFC andone cluster including SMG, AG, and IPL.

Compared to HC, MDD patients showed increased positive FC between cerebellar Crus I and left dlPFC regions. MDD patients had significantly higher positive FC between right dlPFC and right supramarginal gyrus (SMG), angular gyrus (AG), inferior parietal lobule regions (IPL; see Table 2 and Figure 1). We did not detect any group difference of fALFF.

**Table 2:** Regions showing abnormal connectivity with dorsolateral prefrontal cortex in MDD compared to HC.

**ROIs**	**Region**	**L/R**	**Cluster size (Voxels)**	**Peak coordinates**			**Peak intensity**
				**X**	**y**	**z**	
Left dlPFC	Cerebellar Crus I	R	34	24	−81	24	4.67
Right dlPFC	SMG	R	27	51	−45	30	5.68
	AG	R	11				
	IPL	R	8				

*GRF correction for multiple comparison correction with voxel p < 0.001*.

*L, Left; R, Right; dlPFC, Dorsolateral prefrontal cortex; SMG, Supramarginal gyrus; AG, Angular gyrus; IPL, Inferior parietal lobule*.

## Discussion

Clinicians generally accept that MDD patients demonstrate dysfunctional cognition, especially dysfunctional EF in acute phase. However, few of clinicians realize persistent cognition impairment in euthymia (37) because of inconsistent research results, improved subjective experience (38, 39), and a lack of objective cognition tests that are appropriate and can be easily implemented for MDD patients (37). Here, we made a further exploration of cognition performance in remitted and partially remitted MDD patients *via* multiple EF tests and fMRI, in order to reconcile inconsistencies of published literatures and developed the understanding of persistent EF in remitted MDD patients. In addition, our study reminds clinicians to focus on EF in remitted MDD patients.

We found an increased FC between the right dlPFC and right SMG. SMG together with AG forms temporoparietal junction (TPJ) (40). Carter et al. indicated that TPJ is involved in language, memory, attention, and social processing, and the convergent processing in TPJ provide context for behavior (40). Wu et al. suggested that TPJ plays a critical role in the integration of bottom-up and top-down attentional control (41). SMG is close to IPL, which is another region involved in adjust control in FPN and functionally connected with dlPFC (40). Therefore, the increased FC between dlPFC and SMG may suggest a neurocompensatory effect of EF network.

We also found that MDD patients showed an increased FC between the right cerebellar Crus I and left dlPFC than HC. Schmahmann reviewed that patients with cerebellum damage showed persistent EF impairment (42). fMRI studies showed that posterior lobe of cerebellum, especially cerebellar Crus I, was activated during EF tasks (43). At network level, cerebellum is functionally connected to task control network such as FPN and SN (44). fMRI studies further revealed that cerebellum generates error codes and transmit error-related signals to dlPFC, which contributes to rapid, continuous adjustment and control (28, 45). Previous studies reported abnormal function and structure of cerebellum in current MDD. For example, Depping et al. demonstrated bilaterally increased regional cerebellar blood flow (46). Guo et al. found that MDD patients had altered fALFF and FC in cerebellar lobule VI and Crus I (47). Arnone et al. reported gray matter reduction in the cerebellum in MDD patients (48). All these results suggested that there may be an altered error processing in MDD. Disturbed error processing may persist with the remssion of major clinical symptoms. Depping et al. show increased volume of bilateral cerebellum in remitted MDD patients (49). Sun et al. showed an increased regional homogeneity in individuals at risk of depression (27). Consistent with these results, although we did not detect any group difference of fALFF, we found an increased FC between cerebellum and dlPFC in the present study, which may suggest an enhanced processing of bottom-up feedback in remitted or partially remitted MDD patients. In line with the notion, Bijsterbosch et al. indicated that enhanced FC on frontal cortex with cerebellum was associated with supraliminal error correction (50). To our knowledge, other studies reported abnormal FC associated with cognition between cerebellum and other brain region such as temporal cortex (51) and posterior cingulate cortex (52), however, this is the first study that reported an enhanced feedback processing in remitted and partially remitted MDD patients. Therefore, more researches are needed to replicate the result.

Taken together, although MDD patients showed improvement in SVFT, SCWT, WCST, and were comparable with HC on all EF tests after 8-weeks treatment, we still found an increased intrinsic connectivity within the EF network in remitted and partially remitted MDD patients. The result remained true in the case of taking residual depressive symptoms into account, which suggested that EF impairment might be a trait-like characteristic of MDD and fluoxetine only could improve impaired EF to some extent rather than resolve. More generally, conventional antidepressant like selective serotonin re-uptake inhibitors may fail to resolve executive dysfunction of MDD patients. Persistent EF impairment have a negative influence on the social ability and functional recovery (53). Therefore, adjunctive treatment targeting to EF may contribute to maximize the efficacy of routine therapy. Compared to placebo and duloxetine, Mahableshwarkar et al. found that vortioxetine had significant and direct improved effects on EF in MDD patients (54). Similarly, Smith et al. found that vortioxetine increased efficiency of neural circuit supporting EF in remitted MDD patients whose EF performance in comparison with HC, which suggested that vortioxetine had direct and mood-independent effects on EF (25). Since we found neurocompensatory effects, i.e., inefficient neural activity, vortioxetine may be an effective candidate drug for impaired EF of MDD. Erythropoietin is another alternative drug for improving EF. Miskowiak et al. indicated that erythropoietin significantly improved neural correlates of EF and the effect was dissociated from mood symptoms or red blood cells (55). Besides drug therapy, cognitive remediation training and non-invasive neuromodulatory treatment such as transcranial magnetic/direct current stimulation may be a potential candidate therapy for impaired EF (56, 57). Our study reminds clinicians that improvement in the clinical symptoms of MDD patients does not mean remitted EF. It may be necessary to focus on the EF of patients with who are in clinical remission and partial remission, and to adjust the existing treatment strategy to assist with physical therapy or psychotherapy to improve the executive function of patients.

Several limitations of present study should be noted. Firstly, the 8-weeks treatment period is short. Some patients did not achieve full remission and the HAMD score of the MDD group is still higher than HC. We cannot rule out the potential confounding effects of residual symptoms though we add it as covariates. Secondly, the study is a pseudo-longitude study because we did not follow HC. Therefore, we cannot explain the practice and time effects. Thirdly, we used a small sample size because only these patients completed fMRI and all tests we needed at week 8, and we did not assess each aspect of EF yet in the study. Abnormal FC in EF network may due to other components of EF we did not access. Further studies using overall assessment of EF, large MDD sample with long-term treatment, ideally, full recover patients can be an important complement to our study.

## Conclusion

EF impairment is still observed in MDD patients despite the remission or partially remission of depressive symptoms. Clinicians should focus on the residual cognitive symptoms, which contribute to the increased therapeutic effect of antidepressants. Furthermore, enhanced intrinsic FC within EF network may be a potential neural target for intervention of EF.

## Data Availability Statement

The datasets generated for this study are available on request to the corresponding author.

## Ethics Statement

The studies involving human participants were reviewed and approved by Ethical Committee of the First Hospital of Shanxi Medical University. The patients/participants provided their written informed consent to participate in this study.

## Author Contributions

KZ participated in study design and supervision. NS, KZ, and YaW participated in the recruitment, diagnosis, and assessment of patients. AZ and CY participated in the data collection and management. YuW, AZ, GL, and PL participated in the data analysis. YuW wrote the draft and finished the manuscript. AZ, GL, and NS participated in reviewing and editing. All authors contributed and approved the final manuscript.

## Conflict of Interest

The authors declare that the research was conducted in the absence of any commercial or financial relationships that could be construed as a potential conflict of interest.

## Abbreviations

MDD: Major depressive disorder
HCs: Healthy controls
DSM-IV: Diagnostic and Statistical Manual of Mental Disorders - Fourth Edition
HAMD-17: 17-item Hamilton depression rating scale
EF: Executive Function
SVFT: Semantic Verbal Fluency Test
SCWT: Stroop Color Word Test
RRT: Relative Reaction Time
DST: Digital Span Test
WCST: Wisconsin Card Sorting Test
RA: Response Administered
CC: Categories Completed
RC: Correct Response
RE: Errors Response
PE: Perseverative Errors
nPE: non-perseverative errors
fMRI: functional Magnetic Resonance Imaging
ROI: Regions of interest
FC: Functional Connectivity
fALFF: fractional Amplitude of Low-frequency Fluctuations
dlPFC: Dorsolateral prefrontal cortex
SMG: Supramarginal Gyrus
AG: Angular Gyrus
IPL: Inferior Parietal Lobule
GRF: Gaussian Random Field
SPSS 18.0: Statistical Product and Service Solutions 18.0.
